# The Histopathological Spectrum of Pyogenic Granuloma: A Case Series

**DOI:** 10.1155/2016/1323798

**Published:** 2016-06-12

**Authors:** Vinay Marla, Ashish Shrestha, Khushboo Goel, Sajeev Shrestha

**Affiliations:** ^1^Department of Oral Histology & Pathology, College of Dental Surgery, BP Koirala Institute of Health Sciences, Dharan, Sunsari 56700, Nepal; ^2^Department of Periodontics & Implantology, College of Dental Surgery, BP Koirala Institute of Health Sciences, Dharan, Sunsari 56700, Nepal

## Abstract

*Background.* Pyogenic granuloma is a reactive tumor-like lesion commonly affecting the oral cavity. These lesions usually appear as localized solitary nodule with a sessile or pedunculated base and colour varying from red, purplish, or pink, depending on the vascularity of the lesion. Pyogenic granuloma shows predilection for gingiva and is usually slow growing, but at times it shows rapid growth. The natural course of this lesion can be categorized into three distinct phases, namely, (i) cellular phase, (ii) capillary phase/vascular phase, and (iii) involutionary phase. Histopathologically, pyogenic granuloma is classified into lobular capillary hemangioma (LCH) and non-lobular capillary hemangioma (non-LCH).* Case Presentation*. In this series, four cases (varied age groups and both genders) of pyogenic granuloma showing varying histopathological presentation in relation to its clinical course have been described. The lesion in its early phase reveals diffuse endothelial cells, with few budding into capillaries. Among the capillary phase, the LCH type shows numerous blood vessels organized into lobular aggregates whereas the non-LCH type does not show any such organization and resembles granulation tissue. The involutionary phase shows healing of the lesion and is characterized by extensive fibrosis in the connective tissue.* Conclusion.* In conclusion, knowledge of the various histopathological presentation of this lesion is necessary for proper identification.

## 1. Background

Pyogenic granuloma is a commonly occurring reactive lesion of the oral cavity. It is non-neoplastic in nature and is hence referred to as a tumor-like lesion [1]. The term “pyogenic granuloma” was coined by Hartzell in 1904 and is still being used to denote this lesion [2]. But this terminology is confusing since neither it is due to bacterial infection nor does it produce any pus. Also, histopathologically there is no granuloma formation [3].

Pyogenic granuloma involves the gingiva most frequently and presents as a nodular growth which may be slow growing or rapid in nature [1, 4]. The course of the lesion can be described as “early,” “established,” and “healing” type. The colour of the lesion also varies and is dependent on the vascularity of the lesion in relation to its clinical course [5]. The early lesions are usually pinkish in colour and resemble the normal mucosal colour. Established lesions are reddish to purplish due to the increased vascularity whereas the late healing type presents as pinkish to whitish mass. These different phases of pyogenic granuloma can be appreciated on the microscopic level as well [6]. The natural course of this lesion can be categorized into three distinct phases, namely, (i) cellular phase, (ii) capillary phase/vascular phase, and (iii) involutionary phase. Histopathologically, pyogenic granuloma is classified into lobular capillary hemangioma (LCH) &and non-lobular capillary hemangioma (non-LCH) [7]. This case series describes four cases of pyogenic granuloma which depicts the various phases of its clinical course.

## 2. Case Presentation

A total of four cases have been selected for describing the various histopathological presentations. The clinical descriptions of these cases have been described in Table 1.

Clinically, these cases show general features like nodular presentation, showing similar size, occurring more commonly in females and located at gingival and extragingival sites. The histopathological presentation shows variations according to the various phases and has been described below.

**Figure 1:**
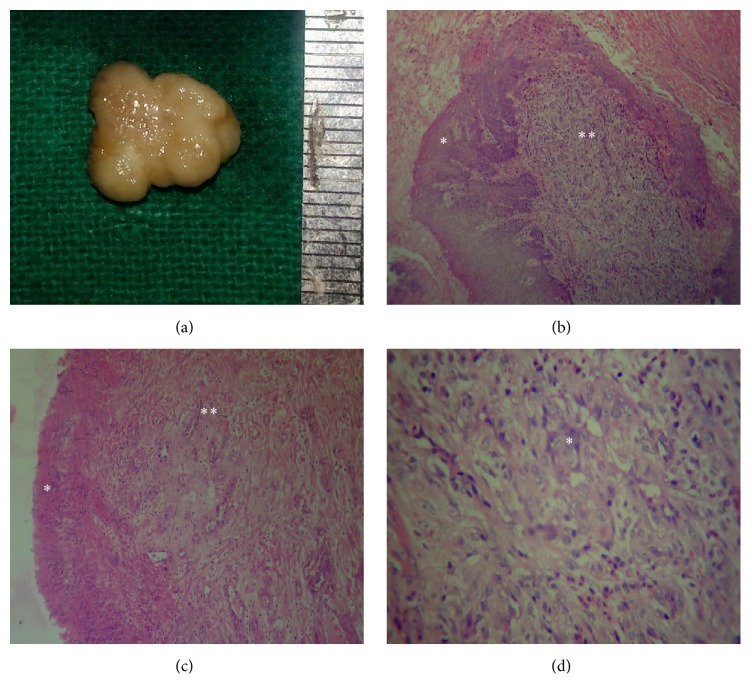
Case  1 description: (a) gross specimen; (b) low power view showing hyperplastic parakeratinized epithelium (*∗*) and underlying cellular stroma (*∗∗*) (H&E, 100x); (c) low power view showing fibrinopurulent membrane (*∗*) and underlying cellular stroma showing few proliferating capillaries (*∗∗*) (H&E, 100x); (d) high power view showing dense aggregation of plump endothelial cells (*∗*) with little evidence of lumen formation (H&E, 400x).

Case 1 shows histopathological features suggestive of the early stage/cellular phase. The gross specimen measured approximately 1 cm and showed a lobular surface (Figure 1(a)). Microscopic evaluation revealed discontinuous hyperplastic parakeratinized stratified squamous epithelium (Figures 1(b) and 1(c)). The underlying connective tissue stroma revealed high cellularity comprising diffuse endothelial cells throughout the stroma with little evidence of any lumen formation (Figure 1(d)).

**Figure 2:**
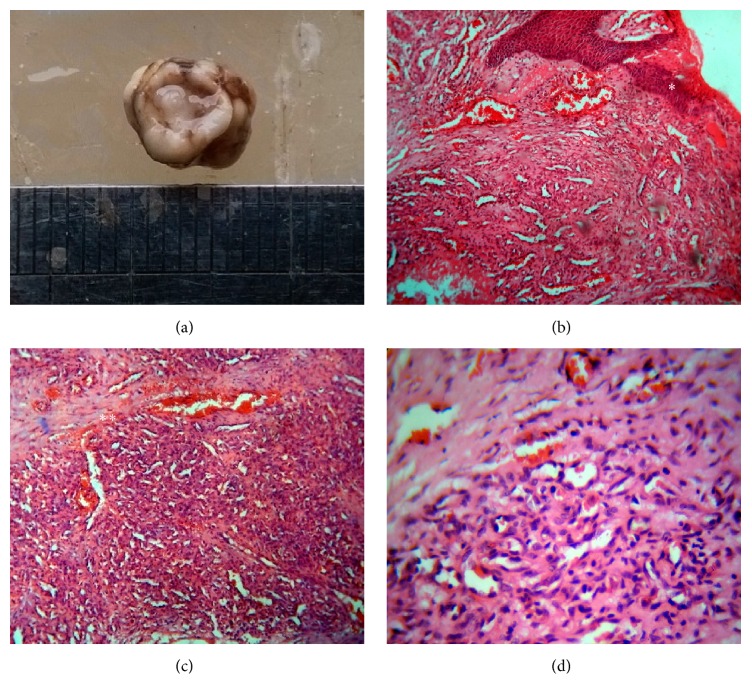
Case  2 description: (a) gross specimen; (b) low power view showing discontinuous parakeratinized stratified squamous epithelium (*∗*) with vascular spaces in the underlying stroma (H&E, 100x); (c) low power view showing numerous capillaries arranged within lobulated spaces (*∗∗*) (H&E, 100x); (d) high power view showing numerous endothelial cell lined capillaries (H&E, 400x).

Case 2 shows features conforming to the established stage of pyogenic granuloma. Gross examination revealed the growth to be less than 1 cm in size (Figure 2(a)). Low power view revealed discontinuous epithelium. The underlying stroma was highly vascular with few engorged capillaries. These vascular areas were arranged as various lobules with peripheral connective tissue septae formation (Figures 2(b) and 2(c)). High power view showed lobular area with numerous plump endothelial cell lined capillaries (Figure 2(d)). These features were suggestive of lobular capillary hemangioma type of pyogenic granuloma.

**Figure 3:**
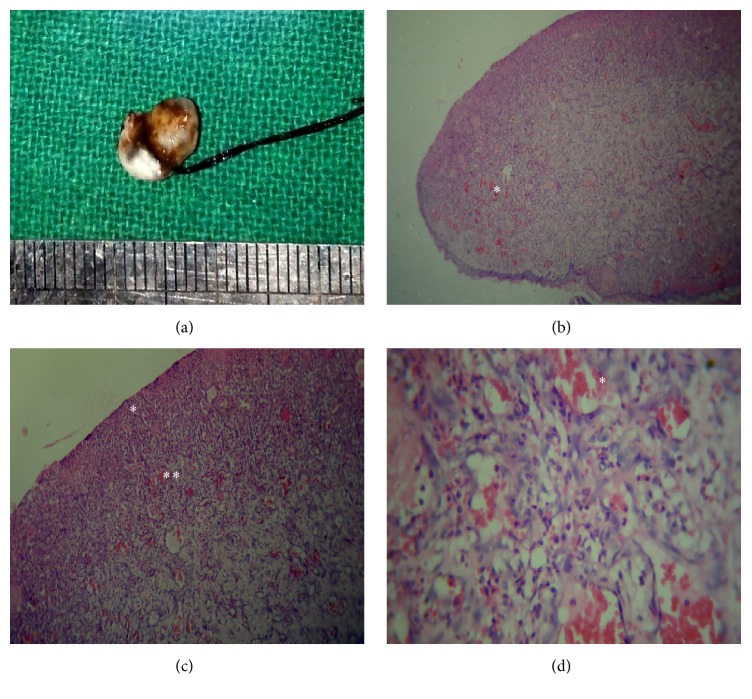
Case  3 description: (a) gross specimen; (b) scanning power view showing numerous endothelial cell lined proliferating capillaries and dense inflammatory cell infiltration (*∗*) (H&E, 50x); (c) low power view showing fibrinopurulent membrane (*∗*) and underlying stroma showing numerous endothelial cell lined capillaries (*∗∗*) (H&E, 400x); (d) high power view showing numerous endothelial cell lined vascular spaces engorged with red blood cells (*∗*) (H&E, 400x).

Case 3 also describes an established stage of pyogenic granuloma. Gross specimen was smaller than 1 cm and revealed haemorrhagic areas (Figure 3(a)). Scanning power showed discontinuous parakeratinized stratified squamous epithelium and underlying stroma revealed numerous vascular spaces (Figures 3(b) and 3(c)). High power view showed numerous dilated capillaries engorged with red blood cells and proliferating capillaries in a loose inflammatory stroma (Figure 3(d)). There was no fibrous septae formation suggesting that this lesion correlated with the non-lobular capillary hemangioma type of pyogenic granuloma.

**Figure 4:**
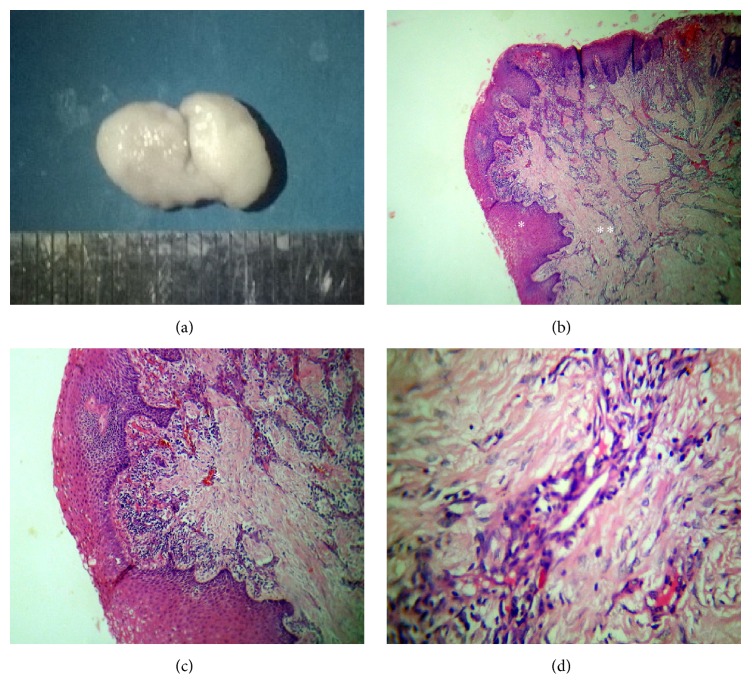
Case  4 description: (a) gross specimen; (b) scanning view showing hyperplastic parakeratinized stratified squamous epithelium (*∗*) and connective tissue with numerous endothelial cell lined capillaries and dense fibrous stroma (*∗∗*) (H&E, 50x); (c) low power view: stroma showing numerous proliferating endothelial cell lined capillaries (*∗*) (H&E, 100x); (d) high power view showing fibrosis around capillaries (H&E, 400x).

Case 4 describes the healing/involutionary phase of pyogenic granuloma. Gross specimen measured roughly 1 cm in size (Figure 4(a)). Microscopic examination revealed discontinuous parakeratinized stratified squamous epithelium (Figures 4(b) and 4(c)). Underlying connective tissue stroma was fibrous and revealed numerous endothelial cell lined capillaries with perivascular inflammatory infiltration and thick bundles of collagen fibres throughout the stroma (Figure 4(d)).

## 3. Discussion

Pyogenic granuloma was first identified by Poncet and Dor in 1897 who described it as a vascularised mass and named it “Human Botryomycosis” [8]. The term pyogenic granuloma is being used now to describe this lesion but it is considered to be a misnomer [3, 9]. Due to its vascularity, the term “Telangiectatic Granuloma” has also been proposed [3].

Low-grade chronic irritation, trauma, and hormonal imbalances are said to be the main etiology for pyogenic granuloma which results in the overzealous proliferation of vascular type of connective tissue [1, 3, 10]. Poor oral hygiene leading to accumulation of plaque and calculus and overhanging restorations are said to be the most common precipitating factors [10]. Other etiological agents include use of certain immunosuppressive drugs and oral contraceptives. Nonspecific bacterial infection is thought to be a secondary involvement rather than being the main etiology of this lesion [11].

Clinically, oral pyogenic granuloma appears as a nodular mass ranging from few millimetres to centimetres in size and are usually slow growing and asymptomatic [9, 11]. These lesions show a striking predilection for gingiva involving the marginal gingiva and interdental papilla commonly. However, it can occur in other sites like lips, tongue, buccal mucosa, and palate [12]. The colour of the lesion varies from pink, purplish, to red and is dependent on the vascularity of the lesion [5]. Pyogenic granuloma occurs frequently during pregnancy especially during the second and third trimesters wherein it is referred to as “pregnancy tumor” [13]. Increased levels of oestrogen and progesterone modify the vascular response to local irritants that lead to the occurrence of the lesion. Histopathologically, pregnancy tumor has features similar to pyogenic granuloma [13, 14].

The microscopic picture of pyogenic granuloma in general shows exuberant granulation tissue which is covered by atrophic/hyperplastic epithelium that may be ulcerated at times and reveals fibrinous exudates. Presence of numerous endothelium-lined vascular spaces and proliferation of fibroblasts and budding endothelial cells are the characteristic features of pyogenic granuloma. Presence of mixed inflammatory cell infiltration is also observed [15]. Cawson et al. have described two variants of pyogenic granuloma depending on the rate of proliferation and vascularity, namely, (i) lobular capillary hemangioma and (ii) non-lobular capillary hemangioma [16]. However, it should be remembered that these terms have been used to describe pyogenic granuloma based on its histopathological variations only and it is not a true hemangioma in the real sense. Hemangiomas are benign tumors consisting of endothelial cell lined blood vessels which can occur within the oral cavity and should be considered as a differential diagnosis for pyogenic granuloma which is actually a reactive lesion [9]. The LCH type of pyogenic granuloma is characterized by proliferating blood vessels organized in lobular aggregates whereas the non-LCH type shows high vascular proliferation resembling granulation tissue [4, 16]. The differences between LCH and non-LCH type have been summarized in Table 2 [17]. This suggests that there might be different evolutionary pathways for both types of pyogenic granuloma [18].

Sternberg et al. suggested three distinct phases to describe the course of pyogenic granuloma. The “early phase” reveals a compact cellular stroma with little lumen formation. The next phase described as the capillary phase reveals lobules which are highly vascular with abundant intraluminal red blood cells. The final phase referred to as “involutionary phase” shows intra- and perilobular fibrosis. This phase is suggestive of healing phase of pyogenic granuloma [19]. Clinical correlation should be done for various phases of pyogenic granuloma, with younger lesions being red to purple due to high vascularity whereas older lesions become collagenized and appear pink [1, 6].

Depending on the different stages of pyogenic granuloma, certain lesions come in as differential diagnosis. Younger lesions may be mistaken for conventional granulation tissue histopathologically or hemangioma, Kaposi's sarcoma, and bacillary angiomatosis clinically [17, 20]. Hemangioma generally presents in extragingival locations and is devoid of any inflammatory components, points which help it in differentiating from pyogenic granuloma [9]. Histopathologically, the absence of atypical cells and bizarre vascular channels helps to differentiate pyogenic granuloma from Kaposi's sarcoma whereas absence of any granular bacterial material differentiates it from bacillary angiomatosis [21]. Similarly older lesions can be mistaken for oral fibroma, peripheral giant cell granuloma, or peripheral ossifying fibroma. Careful histopathological evaluation is necessary to identify this lesion. The older lesions show certain resemblances to oral fibroma or peripheral ossifying fibroma due to the presence of extensive fibrosis in the stroma. However, the increased vascular component and the inflammatory infiltrate are indicative of pyogenic granuloma and helps in differentiating it from these lesions. Also, a prior history of bleeding from the growth would be suggestive of the involutionary phase [3, 11, 22]. Overall, a careful clinical and histopathological correlation is sufficient to identify pyogenic granuloma [1, 3, 4, 9].

## 4. Conclusion

Pyogenic granuloma is a commonly occurring reactive lesion of the oral cavity and is non-neoplastic in nature. Presence of histopathological variation is related to its chronological phase. Knowledge of the same is essential for understanding the lesions. Also clinical correlation should be done for accurate diagnosis.

## Figures and Tables

**Table 1:** Clinical profile of the four cases of pyogenic granuloma.

Sl. number	Age	Gender	Site	Size	Histopathological category
Case 1	40	Female	Gingiva	1-2 cm	Cellular phase
Case 2	40	Female	Buccal mucosa	1-2 cm	Capillary phase, LCH type
Case 3	09	Male	Buccal mucosa	<1 cm	Capillary phase, non-LCH type
Case 4	23	Female	Gingiva	1-2 cm	Involutionary phase

LCH: lobular capillary hemangioma; non-LCH: non-lobular capillary hemangioma.

**Table 2:** Histopathological differences between LCH and non-LCH type of pyogenic granuloma.

LCH type of pyogenic granuloma	Non-LCH type of pyogenic granuloma
Vessels in lobular aggregates	No aggregation, focal fibrous tissue
Proliferating blood vessels	Vascular core resembling granulation tissue
Small luminal diameter	Larger luminal diameter
Perivascular mesenchymal cells *α*SMA positive	Perivascular mesenchymal cells, *α*SMA negative

LCH: lobular capillary hemangioma; non-LCH: non-lobular capillary hemangioma; *α*SMA: *α*-smooth muscle actin.

## Abbreviations

LCH: Lobular capillary hemangioma
Non-LCH: Non-lobular capillary hemangioma
SMA: Smooth muscle actin.
